# The Long-Term Effect of Cancer on Incident Stroke: A Nationwide Population-Based Cohort Study in Korea

**DOI:** 10.3389/fneur.2019.00052

**Published:** 2019-02-05

**Authors:** Hyun-Soon Jang, Jimi Choi, Jaewon Shin, Jong-Won Chung, Oh Young Bang, Gyeong-Moon Kim, Woo-Keun Seo, Juneyoung Lee

**Affiliations:** ^1^Department of Neurology, Samsung Medical Center, School of Medicine, Sungkyunkwan University, Seoul, South Korea; ^2^Department of Biostatistics, College of Medicine, Korea University, Seoul, South Korea; ^3^Department of Digital Health, The Samsung Advanced Institute for Health Sciences & Technology (SAIHST), Sungkyunkwan University, Seoul, South Korea

**Keywords:** cancer, stroke, incidence, propensity score matching, epidemiology, chemotherapy, survival

## Abstract

**Background and Purpose:** Despite the recent growing interest in the cancer–stroke association, the long-term effect, and organ-specific association with stroke incidence in subjects with cancer have not been clearly defined.

**Methods:** Data were obtained from the Korean National Health Insurance Service National Sample Cohort database between 2002 and 2015. To investigate the effects of cancer on stroke incidence, subjects were classified into cancer and non-cancer groups based on the period after cancer diagnosis and origin organ of cancer. To minimize the effects of selection bias, we performed a propensity score matching analysis with covariates of demographic data, vascular risk factors, antithrombotics use and statin use. Incident stroke was diagnosed based on operational definition and classified into ischemic stroke and hemorrhagic stroke.

**Results:** Data of 20,707 subjects with cancer and 675,594 without cancer were analyzed for 7 follow-up years. The subjects with cancer had higher risk of any stroke (subdistribution hazard ratio [SHR], 1.13; 95% confidence interval [CI], 1.02–1.26; *p* = 0.0181) than those without cancer. Similar trend was found for ischemic stroke (SHR, 1.17; 95% CI, 1.05–1.31; *p* = 0.0054), but not for hemorrhagic stroke. The risk of stroke was increased in subjects with cancer in the digestive organ, respiratory and intrathoracic organ, and “others (such as breast and female and male reproductive organs)” in 3 years; however, the association disappeared thereafter except those with “others” cancer. Chemotherapy increased the risk of ischemic stroke (SHR 1.21; 95% CI, 1.03–1.41).

**Conclusions:** Cancer increases the risk of stroke at 3 years after the diagnosis of cancer, and the effect was maintained for 7 years. The association between cancer and stroke incidence depends on the organ from which the cancer originated and chemotherapy.

## Introduction

Thromboembolism, a well-known complication affecting 7–18% of cancer patients, is among the major causes of death (1–4). Traditionally, venous thrombosis, affected by Virchow's triad-related thrombosis, was identified as a major thromboembolic complication of cancer (3, 5). Arterial thrombosis in cancer patients was recently highlighted, and stroke is among its most important forms (6–12). In an autopsy series from 1985, 14.6% of cancer patients had pathological evidence of cerebrovascular complications (13). Cancer-related hypercoagulability can contribute to the development of stroke, and stroke with and without cancer appears to have different mechanisms, clinical manifestations, progression, and prognoses (6, 14). However, only a few population-based studies have investigated the cancer–stroke relationship, and debate persists on the long-term or organ-specific effect of cancer on stroke incidence. Prior studies suggested that incident cancer was associated with an increased short-term risk of stroke; however, findings were mostly based on short-term observations (6, 7, 10, 15–19).

Therefore, we investigated the effects of cancer on stroke incidence over a 7 year period considering the time from the index cancer diagnosis and cancer location using data from a nationwide cohort, the Korean National Health Insurance Service National Sample Cohort (NHIS-NSC) database, which represents the entire Korean population (20).

## Methods

### Study Design and Source of Data

The study analyses used the NHIS-NSC database in South Korea, which contains a population-based cohort of >1,000,000 subjects established by the NHIS (20). Because all Korean medical organizations are compulsorily linked by NHIS and NHIS is a universal health care system of South Korea, NHIS database includes all information on ~50 million whole Korean residence. The NHIS-NSC database was made to represent NHIS database by being stratified into 1,476 strata using age group, sex, participant's eligibility status, and income level. A systematic stratified random sampling was conducted to maintain a sampling rate of 2.2% of the total eligible Korea population in 2002 and followed up for 13 years until 2015. Because the main purpose of the NHIS-NSC was academic interest in terms of system efficacy and relevance to public health and medical research, it is appropriate to investigate cancer by the incidence of stroke.

As NHIS-NSC database is a publicly opened data, the institutional review board of our institution approved the waive of reviewing this study (SMC 2016-07-168).

### Original Population Subjects

We selected the target population among those who were included in the NHIS-NSC database between January 1, 2006, and December 31, 2008 (*N* = 1,041,441). The inclusion criterion was the presence of at least one NHIS record during that period. Among them, patients aged <20 years, those who had a history of cancer or stroke between 2002 and 2005, or those who died at the index year and month were excluded. The eligible subjects were classified into a cancer group and a non-cancer group.

Subgroup analyses were performed to investigate the effects of smoking because, in the NHIS-NSC, the smoking status data are available for the subgroup who participated in the health promotion program.

### Operational Definitions

The cancer diagnosis was based on the C00–C97 codes of the International Classification of Disease 10th edition (ICD-10) and V193 code of the Health Insurance Review and Assessment Service. Cancer group was subdivided according to the original organ location of the cancer: “lip, oral cavity, and pharynx”; “digestive organs”; “respiratory and intrathoracic organs”; and “others.” NHIS in Korea does not provide information about detailed cancer originated from genital organ or breast due to sensitive personal information issue.

For the diagnosis of stroke, we developed an appropriate operational definition (because the use of the ICD-10 diagnosis alone is insufficient) as follows: those who had an ICD-10 code of I60–I63, who had been admitted for ≥3 days, and who underwent computed tomography (CT) or magnetic resonance imaging (MRI) in the brain (21). Subjects who underwent acute recanalization therapy using intravenous tissue plasminogen activator infusion or endovascular treatment were considered to have ischemic stroke, even if they failed to meet the criteria.

Vascular risk factors used in this study included hypertension, diabetes mellitus, dyslipidemia, coronary artery disease, heart failure, and atrial fibrillation. The detailed operational definitions used in this study are provided in the Supplementary Materials. In brief, the diagnosis of hypertension, diabetes mellitus, or dyslipidemia was made based on ICD-10 codes or history of medication use. The diagnosis of atrial fibrillation, heart failure, or coronary artery disease was based on ICD-10 codes.

To determine the independent association between cancer and stroke, details of anticoagulant, antiplatelet agent, and statin use before stroke onset were also obtained. We classified the cancer group into those treated by chemotherapy and those not-treated by chemotherapy to investigate the effect of chemotherapy in stroke risk. We used drug classification code according to purpose which was provided by Korean Health Insurance Review and Accessment Service (HIRA). The list is composed of 2,949 anti-cancer drugs (based on drug product name) including both angiogenesis inhibitors and hormone agents. To clarify the association between chemotherapy and stroke incidence, only administration of anti-cancer drug before the onset of stroke was considered as chemotherapy group among stroke group.

### Statistical Analysis

The primary objective of this study was to examine the difference in stroke incidence between the cancer and non-cancer groups. To do this, a patient's index date was defined as the date of the first diagnosis in the cancer group, whereas those in the non-cancer group was the date of the first visit for medical attention after January 1, 2006. In both groups, events within 7 years were captured until the end of the study, which was defined as the last visit in the past 7 years from the index date. As death is a common competing risk in patients with cancer, a risk survival analysis was performed to calculate the cumulative stroke incidence in both groups and then compared using Gray's test for an equality of cumulative incidence functions. Meanwhile, the relative risk of stroke was assessed using a proportional hazard model for subdistribution proposed by Fine and Gray with a competing risk in the analysis. To improve the analytical robustness, we considered both unadjusted and adjusted models with covariates (sex, age group, diabetes mellitus, hypertension, coronary artery disease, heart failure, atrial fibrillation, and medication use). The baseline characteristics of the study subjects are presented as frequency and proportion, whereas comparisons of the proportions between cancer and control groups were performed using the chi-square test. To minimize the effects of selection bias, we performed a propensity score matching analysis with a 1:1 intergroup ratio. The propensity score was calculated using a multiple logistic regression model with covariates of sex, age group, hypertension, diabetes mellitus, dyslipidemia, coronary artery disease, heart failure, atrial fibrillation, and medication use (anticoagulant, antiplatelet agent, statin). Matching for propensity score was performed by 5 Greedy methods using OneToManyMATHCH algorithm (http://www2.sas.com/proceedings/sugi29/165-29.pdf). Among these matched-pair data, we performed a stratified subdistribution hazard analysis, conditional logistic regression analysis, or Bowker's test as appropriate. All statistical analyses were conducted using the Statistical Analysis System (SAS) software version 9.4 (SAS Institute, Cary, NC, United States), and statistical significance was considered at two-sided *p* < 0.05.

## Results

**Figure 1:**
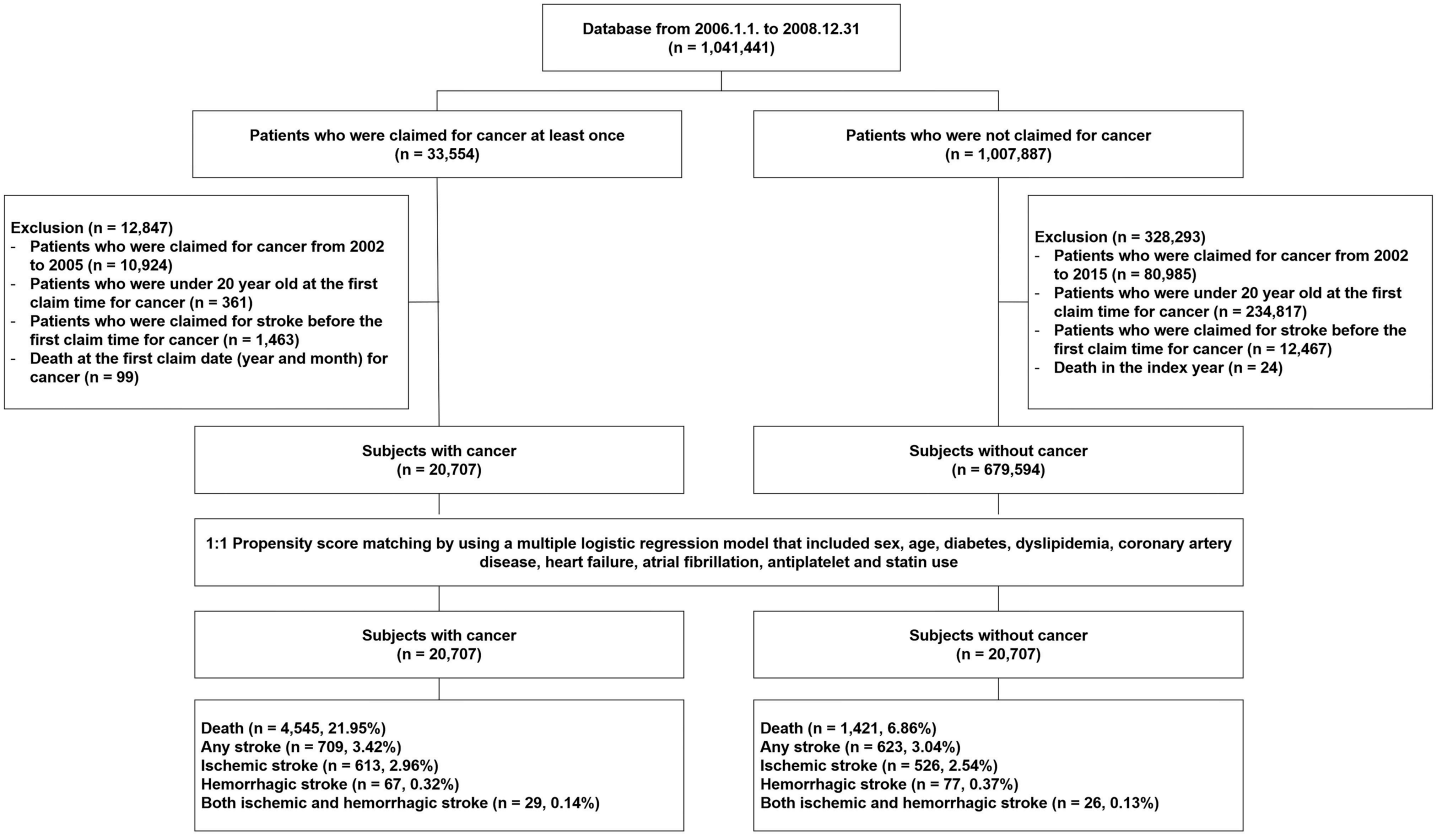
Selection of study subjects from the National Health Insurance Service National Sample Cohort database of South Korea.

Between January 1, 2006, and December 31, 2008, a total of 1,041,441 subjects were included in the NHIS-NSC database. With the application of our exclusion criteria mentioned in the Methods section, a total of 20,707 subjects with cancer and 675,594 subjects without cancer remained in the analysis (Figure 1). Patients in the cancer group were predominantly older than those in the non-cancer group, and male patients were also predominant (Table 1). The vascular risk factors tested in this study were more prevalent in the cancer group than those in the non-cancer group. Significant intergroup differences in age, sex, comorbidities, concurrent medication, and follow-up time were also found (Table 1). Mean follow-up duration of cancer group was 69.7 ± 27.3 months and that of non-cancer group was 82.7 ± 8.0 months.

**Table 1:** Comparison of baseline characteristics between subjects with and without cancer.

	**Original population cohort**			**Matched cohort**		
	**Cancer group (*n* = 20,707)**	**Non-cancer group (*n* = 679,594)**	***P*^***a***^**	**Cancer group (*n* = 20,707)**	**Non-cancer group (*n* = 20,707)**	***P***
Age group, y (%)			<0.0001			0.9994 ^*a*^
19–29	847 (4.1)	187,646 (27.6)		847 (4.1)	846 (4.1)	
30–39	2,122 (10.2)	168,142 (24.7)		2,122 (10.2)	2,124 (10.3)	
40–49	4,079 (19.7)	150,510 (22.1)		4,079 (19.7)	4,078 (19.7)	
50–59	4,495 (21.7)	88,528 (13.0)		4,495 (21.7)	4,498 (21.7)	
60–69	4,613 (22.3)	50,237 (7.4)		4,613 (22.3)	4,614 (22.3)	
70–79	3,373 (16.3)	24,964 (3.7)		3,373 (16.3)	3,372 (16.3)	
80+	1,178 (5.7)	9,567 (1.4)		1,178 (5.7)	1,175 (5.7)	
Sex			<0.0001			>0.9999 ^*b*^
Male, *n* (%)	10,575 (51.1)	335,736 (49.4)		10,575 (51.1)	10,575 (51.1)	
**COMORBIDITIES**^**†**^, ***n*** **(%)**						
Hypertension	12,685 (61.3)	207,021 (30.5)	<0.0001	12,685 (61.3)	12,682 (61.2)	0.9700 ^*b*^
Diabetes	13,071 (63.1)	212,067 (31.2)	<0.0001	13,071 (63.1)	13,068 (63.1)	0.3719 ^*b*^
Dyslipidemia	14,738 (71.2)	306,923 (45.2)	<0.0001	14,738 (71.2)	14,745 (71.2)	0.0544 ^*b*^
Coronary artery disease	8,197 (39.6)	103,625 (15.2)	<0.0001	8,197 (39.6)	8,196 (39.6)	0.7064 ^*b*^
Heart failure	2,703 (13.1)	26,656 (3.9)	<0.0001	2,703 (13.1)	2,703 (13.1)	>0.9999 ^*b*^
Atrial fibrillation	1,488 (7.2)	13,937 (2.1)	<0.0001	1,488 (7.2)	1,488 (7.2)	>0.9999 ^*b*^
**CONCURRENT MEDICATION**^**‡**^, ***n*** **(%)**						
Anticoagulant	759 (3.7)	6,958 (1.0)	<0.0001	759 (3.7)	703 (3.4)	0.1049 ^*b*^
Antiplatelet agent	3,427 (16.5)	42,612 (6.3)	<0.0001	3,427 (16.5)	3,425 (16.5)	0.4235 ^*b*^
Statin	7,755 (37.5)	147,292 (21.7)	<0.0001	7,755 (37.5)	7,752 (37.4)	0.2150 ^*b*^
**CHEMOTHERAPY**, ***n*** **(%)**						
Ever	6,211 (30.0)			6,211 (30.0)		
Never	14,496 (70.0)			14,496 (70.0)		
Follow-up time, mo			<0.0001			
Mean (SD)	69.7 (27.3)	82.7 (8.0)				
Median (min, max)	84.0 (0.03, 84.00)	84.0 (0.03, 84.00)				

^*^Propensity scores were calculated using a multiple logistic regression model that included sex, age, diabetes, dyslipidemia, coronary artery disease, heart failure, atrial fibrillation, and antiplatelet and statin use.

† Cases that were claimed for the corresponding disease at least once until the event

‡ *Cases that were claimed for the corresponding drug more than twice a month until the event*.

a *p-value by Bowker's test for symmetry*.

b *p-value by conditional logistic regression analysis*.

### Comparison of Stroke Incidence Between Cancer and Non-cancer Groups in the Original Cohort

During the study period, subjects in cancer group had 613 (2.96%) ischemic strokes, 67 (0.32%) hemorrhagic strokes, and 29 (0.14%) both ischemic and hemorrhagic strokes, respectively. In contrast, those in the non-cancer group had 526 (2.54%) ischemic strokes, 77 (0.37%) hemorrhagic strokes, and 26 (0.13%) both ischemic and hemorrhagic strokes, respectively. The cumulative incidences for any, ischemic, and hemorrhagic strokes and death were higher in the cancer group than in the non-cancer group on univariate analysis (Table 2 and Supplemental Table 1). The significant difference was maintained for any stroke (hazard ratio [HR], 1.35; 95% confidence interval [CI], 1.25–1.46; *p* < 0.0001; subdistribution HR with death as a competing event, 1.09; 95% CI, 1.00–1.18; *p* = 0.0424) and ischemic stroke (HR, 1.39; 95% CI, 1.28–1.51; *p* < 0.0001; subdistribution HR with death as a competing event, 1.13; 95% CI, 1.03–1.23; *p* = 0.0076) in covariate-adjusted models with or without the consideration of death as a competing event. However, the difference in hemorrhagic stroke failed to reach statistical significance in the covariate-adjusted models.

**Table 2:** Cumulative incidence and relative hazard of each event in subjects with and without cancer at the end-of-study (after 7 years).

**Event**	**Cumulative incidence at 7 years, % (CI)**			**Hazard ratio (CI)**			
	**Cancer group**	**Non-cancer group**	***P*^***a***^**	**Cause-specific HR**	***p***	**Subdistribution HR^**†**^**	***P***
**ORIGINAL COHORT**	**(*****n*** **=** **20,707)**	**(*****n*** **=** **679,594)**					
Any stroke^*^	3.43 (3.18, 3.68)	1.07 (1.05, 1.10)	<0.0001	1.35 (1.25, 1.46)	<0.0001	1.09 (1.00, 1.18)	0.0424
Ischemic stroke	3.10 (2.87, 3.35)	0.91 (0.89, 0.93)	<0.0001	1.39 (1.28, 1.51)	<0.0001	1.13 (1.03, 1.23)	0.0076
Hemorrhagic stroke	0.46 (0.38, 0.57)	0.21 (0.20, 0.22)	<0.0001	1.15 (0.93, 1.41)	0.2082	0.87 (0.71, 1.08)	0.2138
Death	21.96 (21.40, 22.53)	2.03 (2.00, 2.06)	<0.0001	4.84 (4.68, 5.02)	<0.0001		
**MATCHED COHORT**	**(*****n*** **=** **20,707)**	**(*****n*** **=** **20,707)**					
Any stroke^*^	3.43 (3.18, 3.68)	3.04 (2.81, 3.28)	0.0220	1.29 (1.16, 1.43)	<0.0001	1.13 (1.02, 1.26)	0.0181
Ischemic stroke	3.10 (2.87, 3.35)	2.67 (2.45, 2.89)	0.0069	1.33 (1.19, 1.49)	<0.0001	1.17 (1.05, 1.31)	0.0054
Hemorrhagic stroke	0.46 (0.38, 0.57)	0.50 (0.41, 0.60)	0.6239	1.07 (0.81, 1.41)	0.6327	0.93 (0.71, 1.23)	0.6246
Death	21.96 (21.40, 22.53)	6.87 (6.53, 7.22)	<0.0001	3.60 (3.42, 3.80)	<0.0001		

* *Based on the first occurred event between ischemic and hemorrhagic stroke*.

† *All-cause mortality was considered as a competing risk event*.

*The model was adjusted by sex, age, hypertension, diabetes mellitus, dyslipidemia, coronary artery disease, heart failure, artrial fibrillation, and medication use (anticoagulant, antiplatelet agent, statin)*.

a *p-value by Gray's test for an equality of cumulative incidence functions*.

In the subgroup analysis of subjects with data about smoking status, no significant interaction was observed between smoking status and cancer for ischemic stroke (*p* for interaction = 0.7347; Supplemental Table 2).

### Comparison of Stroke Incidence Between Cancer and Non-cancer Groups in Propensity Score-Matched Cohort

**Figure 2:**
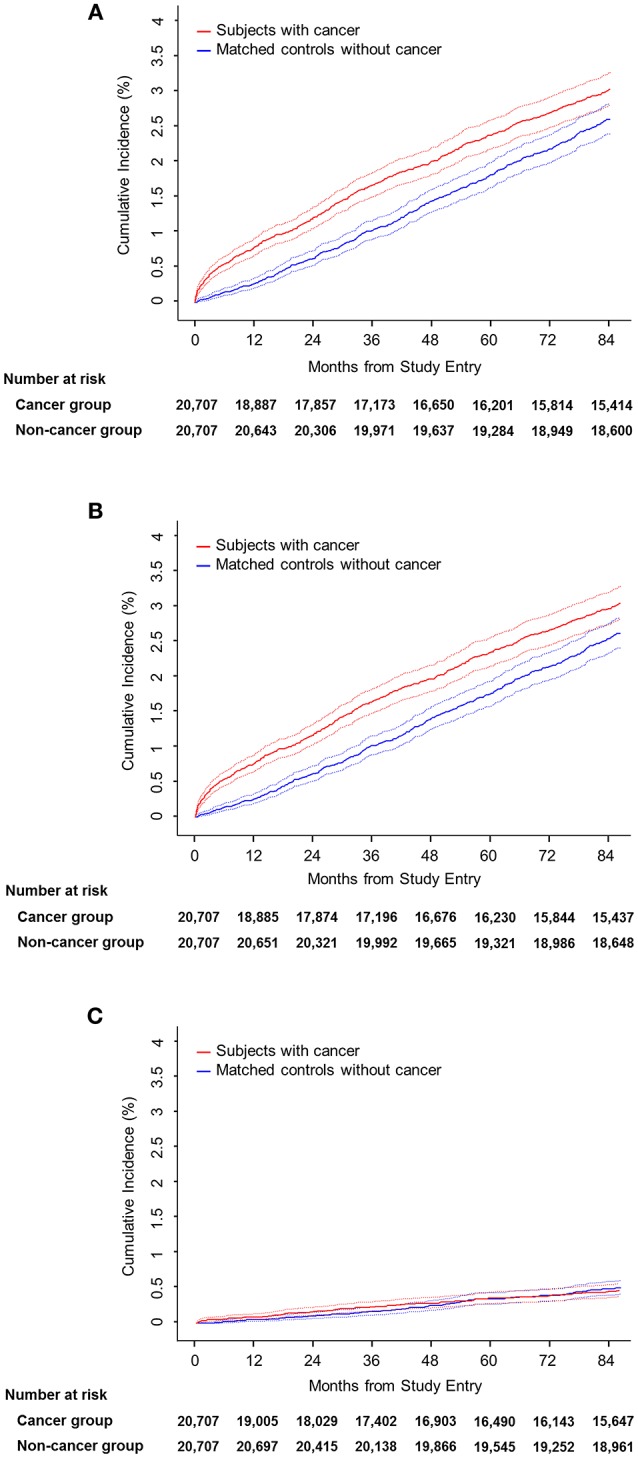
Cumulative incidence by stroke subtype. **(A)** Any stroke. **(B)** Ischemic stroke. **(C)** Hemorrhagic stroke.

A total of 20,707 pairs of cancer and non-cancer subjects were selected by propensity score matching. The baseline characteristics were well-balanced after matching (Table 2). During the 7 year follow-up, a total of 709 strokes (crude rate, 3.42) occurred in the cancer group vs. 629 strokes (crude rate, 3.04) in the non-cancer group, the difference being statistically significant (Figure 2). The hazard ratio of any stroke occurrence in the cancer group was highly significant in both univariate (HR, 3.43; 95% CI, 3.18–3.68; *p* < 0.0001) and multivariate analyses (HR, 1.29; 95% CI, 1.16–1.43, *p* < 0.0001). Furthermore, a significant statistical difference was observed in an adjusted model with a competing risk analysis (subdistribution HR, 1.13; 95% CI, 1.02–1.26; *p* = 0.0181). Ischemic stroke showed a similar pattern of association with the cancer group (subdistribution HR, 1.17; 95% CI, 1.05–1.31; *p* = 0.0054 for ischemic stroke). However, hemorrhagic stroke was not associated with cancer as observed in the analysis of the original population cohort.

### The Association Between Cancer and Stroke According to Time From the Diagnosis of Cancer

In the original cohort, the significant intergroup difference in the relative HR for any and ischemic strokes was maintained during the first 3 years (Supplemental Table 3). However, the relative HR for hemorrhagic stroke was not different except in the second year.

The trends were the same in matched cohort. The significant effects of cancer on the occurrence of any or ischemic stroke was maintained for 3 years and 1 year, respectively. Hemorrhagic stroke was not affected by cancer from the first year after the diagnosis.

### Differential Effects of Cancer on Stroke Occurrence by Originating Organ Distribution

After the 7 years follow-up, the digestive organs (C15–C26) were most frequently affected by cancer, followed by the group categorized as “others” (C50–C58) and respiratory/intrathoracic organs (C30–C39). During the total follow-up period, only “others” showed a significant difference in ischemic stroke compared to the matched controls. However, considering the elapsed time after cancer diagnosis, the digestive organs (C15–C26), respiratory/intrathoracic organs (C30–C39), and “others” categories were significantly associated with the occurrence of ischemic stroke in the first year (Table 3 and Supplemental Table 4).

**Table 3:** Risk of stroke based on time since first cancer diagnosis.

**Organ**	**Total**		**0–1 year**		**1–2 years**		**2–7 years**	
	SubHR^*^ (95% CI)	***P***	SubHR^*^ (95% CI)	***P***	SubHR^*^ (95% CI)	***P***	SubHR^*^ (95% CI)	***P***
**ISCHEMIC STROKE**								
Lip, oral cavity, and pharynx	1.02 (0.69, 1.51)	0.9237	1.34 (0.30, 5.97)	0.7055	0.83 (0.35, 2.02)	0.6880	1.11 (0.70, 1.74)	0.6616
Digestive organs	1.13 (0.96, 1.33)	0.1510	3.33 (2.07, 5.37)	<0.0001	0.98 (0.60, 1.60)	0.9305	1.06 (0.87, 1.30)	0.5605
Respiratory/intrathoracic organs	1.29 (0.93, 1.78)	0.1304	5.03 (2.23, 11.35)	<0.0001	2.31 (0.92, 5.79)	0.0748	0.88 (0.57, 1.35)	0.5481
Thyroid/other endocrine glands	1.00 (0.45, 2.23)	>0.9999	1.00 (0.14, 7.11)	0.9993	1.01 (0.14, 7.17)	0.9931	1.00 (0.38, 2.68)	0.9940
Others	1.24 (1.02, 1.50)	0.0293	2.37 (1.44, 3.90)	0.0007	1.76 (1.04, 2.99)	0.0370	1.09 (0.87, 1.38)	0.4455
**HEMORRHAGIC STROKE**								
Lip, oral cavity, and pharynx	1.33 (0.30, 5.97)	0.7074	–	–	1.39 (0.31, 6.23)	0.6662		
Digestive organs	0.82 (0.55, 1.24)	0.3547	1.00 (0.32, 3.10)	>0.9999	1.58 (0.60, 4.16)	0.3510	0.79 (0.48, 1.31)	0.3638
Respiratory/intrathoracic organs	0.81 (0.39, 1.70)	0.5838	6.01 (0.72, 49.84)	0.0969	2.46 (0.22, 27.09)	0.4633	0.47 (0.17, 1.32)	0.1525
Thyroid/other endocrine glands	1.00 (0.20, 4.96)	0.9994	–	–	1.01 (0.14, 7.13)	0.9963		
Others	1.13 (0.69, 1.86)	0.6155	1.33 (0.30, 5.96)	0.7062	2.14 (0.54, 8.57)	0.2815	1.10 (0.63, 1.94)	0.7358

*SubHR, subdistribution hazard ratio*.

* *Reference group is matched control*.

### Comorbid Stroke and the Risk of Death in the Cancer Group

The cumulative risk of death at 7 years was higher in subjects with any, ischemic, and hemorrhagic strokes than those without (Table 4). The relative risk of death in subjects with stroke at 7 years was 1.21 (1.08–1.37) for any stroke, 1.22 (1.08–1.38) for ischemic stroke, and 1.40 (1.06–1.85) for hemorrhagic stroke in the adjusted model.

**Table 4:** Effects of stroke on survival in patients with cancer.

**Stroke**	**Events**	**Cumulative events at 7 years, *n* (%)**	p^*^	**Hazard ratio (95% CI)**			
				**Univariate HR^**†**^**	***p***	**Multivariate^***a***^^†^**	***p***
Any stroke^*^	Yes (*n* = 709)	307 (43.3)	<0.0001	1		1	
	No (*n* = 19,998)	4,545 (22.7)		1.85 (1.65–2.08)	<0.0001	1.21 (1.08–1.37)	0.0016
Ischemic stroke	Yes (*n* = 642)	275 (42.8)	<0.0001	1		1	
	No (*n* = 20,065)	4,577 (22.8)		1.95 (1.73–2.20)	<0.0001	1.22 (1.08–1.38)	0.0020
Hemorrhagic stroke	Yes (*n* = 96)	50 (52.1)	<0.0001	1		1	
	No (*n* = 20,611)	4,802 (23.3)		2.37 (1.79–3.13)	<0.0001	1.40 (1.06–1.85)	0.0186

a *Model adjusting by age, gender, heart failure, atrial fibrilation, anticoagulant, and antiplatelet use*.

* *p-value by log-rank test*.

† *p-value by Wald test for hazard ratio*.

### The Association Between Chemotherapy and the Risk of Stroke in Subjects With Cancer

Among the subjects with cancer, 6,211 (30.0%) had been administered with chemotherapeutic agents at least once. The distribution of vascular risk factors between those with and without chemotherapy groups according to age group is presented in Supplemental Table 5. In Cox's model to investigate the association between chemotherapy and the risk of stroke, the chemotherapy group showed higher risk of any stroke (subdistribution HR 1.21, 95% CI 1.03–1.41) and ischemic stroke (subdistribution HR 1.19, 95% CI 1.01–1.40) in the adjusted models (Table 5). However, the risk of hemorrhagic stroke was not different between the two groups (subdistribution HR 1.27, 95% CI 0.84–193).

**Table 5:** Effects of chemotherapy on the risk of stroke in patients with cancer.

**Stroke**	**Unadjusted hazard ratio (95% CI)**				Adjusted hazard ratio (95% CI)^*^			
	**Cause-specific HR^**†**^**	***p***	**Subdistribution HR^**†**^**	***p***	**Cause-specific HR^**†**^**	***p***	**Subdistribution HR^**†**^**	***p***
Any stroke	1.57 (1.34–1.83)	<0.0001	1.27 (1.08–1.48)	0.0029	1.43 (1.22–1.67)	<0.0001	1.21 (1.03–1.41)	0.0186
Ischemic stroke	1.53 (1.30–1.80)	<0.0001	1.23 (1.05–1.45)	0.0121	1.40 (1.19–1.65)	<0.0001	1.19 (1.01–1.40)	0.0426
Hemorrhagic stroke	1.76 (1.17–2.67)	<0.0001	1.40 (0.93–2.12)	0.1092	1.56 (1.03–2.37)	0.0359	1.27 (0.84–1.93)	0.2553
Death	3.51 (3.31–3.72)	<0.0001			2.91 (2.74–3.08)	<0.0001		

* *Model adjusted by age, sex, hypertension, diabetes mellitus, dyslipidemia, coronary artery disease, atrial fibrilation, and medication use (anticoagulant, antiplatelet agent, statin)*.

† *All-casue mortality was considered as a competing risk event*.

*p-value by Wald test for hazard ratio*.

## Discussion

In this nationwide cohort study, the cumulative incidence, and relative hazard for ischemic stroke were higher in the cancer than non-cancer group in Korean population. The higher risk for stroke in the cancer group was maintained for 3 year since the diagnosis of cancer, and this early difference in risk remained constant up to 7 years. The co-morbid cancer with stroke resulted in higher risk of death than those without stroke. Besides, the history of chemotherapy was independently associated with the incident stroke.

Recently, a growing body of evidence regarding the association between cancer and stroke has been accumulated. However, most of studies are limited to comparisons between the vascular risk factors or stroke patterns and speculation of its pathogenesis. Although several epidemiological approaches have been attempted, they covered only specific cancer type or featured short follow-up durations (6–8, 15–19, 22, 23). Currently, only two epidemiological studies covered all kinds of cancer. Navi et al. reported the positive association between incident cancer and increased short-term risk of stroke with USA patients (10). In this population-based study using Medicare claims data, lung, pancreatic, colorectal, and breast cancers significantly increased the 3 month cumulative risk of stroke. However, the cumulative risk of stroke was not different between subjects with and without cancer after 1 year. Another epidemiologic study analyzed an individual-level registry in Sweden, which also showed higher risk of ischemic stroke during the first 6 months after the diagnosis of cancer (8). The higher relative risk decreased after 6 months, but the cumulative risk of stroke remained constant at the end of the 10 year follow-up. Our results showed similar trend in a Swedish study. The cumulative risk of ischemic stroke maintained the significance at the end of the 7 year follow-up. Furthermore, the association was consistent in the adjusted model for vascular risk factors, smoking status, and anti-thrombotic use and in competing risk analysis considering death.

As in the previously described studies, the association between cancer and stroke incidence was time-dependent. The higher risk for ischemic stroke in cancer group persisted until 3 years. However, the difference in the risk of stroke disappeared between subjects with and without cancer at each period after 3 years. Several speculations address the cause of the association between cancer and ischemic stroke that was apparent only during the early period after the diagnosis.

First, tumor-related hypercoagulability could be attributed to the occurrence of stroke because early cancer should have a high tumor burden. Therefore, the finding that increased stroke risk until 3 years may reflect that most cancer patients either have achieved cure or long-term remission or have died by 3 years. Second, in a study that used population-based claims data from the Taiwan NHI, after the cancer diagnosis, the increased stroke risk was the greatest within the first 6 months. The authors conjectured that the high fatality rate in the late stage could have resulted in the higher stroke risk at the early period after the diagnosis (7). However, in our study, the cancer group were at a higher risk of stroke after the consideration of death as competing risk, which suggests an independent association from death. Third, chemotherapy for cancer could be associated with the occurrence of stroke (22–26). Chemotherapy is commonly adjusted in the early cancer stage. Accordingly, several studies reported that the cytostatic drug use increases the thromboembolic phenomena (27, 28). This supports the effects of anti-cancer therapy being the greatest during this time period (29, 30). One systematic review also showed that chemotherapy in the cancer group was independently associated with stroke incidence (18). However, two studies reported that stroke incidence was not associated with chemotherapy (6, 19). The results of this study support the hypothesis that chemotherapy can produce increased risk of stroke. We agree that a cautious approach to interpret the positive association between chemotherapy and incidence of stroke is needed because of the lack of cancer stage information. However, despite the limitation, the risk of ischemic stroke was associated with chemotherapy group even after the adjusted model with competing risk analysis.

Another interesting finding was that the risk of ischemic stroke was limited to the specific cancer type. Previous studies reported that lung (7, 10), colorectal (10), pancreatic (10), central nervous system (8), stomach (19), and ovary (18) cancers were associated with increased risk of stroke. However, breast cancer was not associated with stroke (10, 15). This study also showed that the association between cancer and stroke was organ-specific, namely, cancers from digestive organ, respiratory and intrathoracic organs, and others increased the risk ischemic stroke, while thyroid/other endocrine, lip, oral cavity, and pharyngeal cancers did not elevate the risk of stroke with trends similar to the findings of Navi et al. (10). This organ-specific association between cancer and stroke could be partly explained by the histologic type of cancer. Previous studies reported that the risk of thrombotic events in cancer depended on the histologic type, e.g., adenocarcinoma, could promote the coagulation by mucin production (31). Considering that adenocarcinoma frequently originated in the digestive tract, reproductive organs, and respiratory organs, the organ-specific association between cancer and stroke noted here could be due to a different distribution of histologic types based on the organ location.

In this study, the occurrence of cancer was differentially associated with stroke, showing that only ischemic stroke was increased by the occurrence of cancer, whereas hemorrhagic stroke was not. Previous studies reported conflicting data. In Taiwan National Health Insurance, lung cancer and head and neck cancer increased the risk of hemorrhagic stroke (6, 7). However, the Medicare claim data in the USA showed increased risk of hemorrhagic stroke until 6 month from the diagnosis of cancer and then the difference disappeared (10). Our data were consistent with USA data in that the risk of hemorrhagic stroke elevated during the second 2 years but thereafter no difference of hemorrhagic stroke was observed between the subjects with and without cancer.

This study has several limitations. First, as this study was based on medical insurance claims database (NHIS-NSC), the diagnostic accuracy of cancer or stroke cannot be guaranteed. In South Korea, all cancer patients are registered in the National Cancer Registration and Statistics system, and a governmental reimbursement policy for cancer patients is specifically regulated by the NHIS, which can improve the cancer diagnosis reliability. In terms of stroke diagnosis, we aimed to improve the diagnostic reliability by excluding patients who had a stroke history of at least 5 years, which minimizes the possibility of carrying the previous diagnosis of stroke. Furthermore, an operational definition was used for the diagnosis of stroke. Similar strategies were used in previous studies that used NHIS-NSC database data (32). Second, the NHIS-NSC database cannot provide the information on cancer stage at the time of diagnosis. Therefore, we considered death as a competing risk to minimize the effect of cancer stage. Moreover, in order to assess the pure impact of cancer on stroke incidence, we excluded subjects who died during the index year because they might have a fatal non-cancer disease that can affect the incidence of stroke in both cancer and non-cancer groups. Considering that excluding those with advanced cancer in index year would reduce the impact of cancer on the incidence of stroke, we regarded this exclusion as a conservative approach to assessing the main purpose of this study. However, the exclusion raises a possibility of selective bias. Third, we did not obtain data on stroke etiology. However, we considered most of the major vascular risk factors that could reflect part of the etiologic background. Furthermore, we also adjusted for antithrombotic and statin treatment after the diagnosis of stroke. In addition, we performed propensity score matching analyses to reduce a possibility of selection bias although this analysis cannot eliminate the effect of all hidden biases. Forth, a difference of hazard ratio in different models contains a concern of unadjusted confounders. Therefore, to reveal the association more clearly, we performed propensity score matching which could minimize the possibility of selection bias.

In despite of those limitations, several strong points enhance the value of this study. This is the first report revealing the significant association between cancer and stroke incidence for the entire population in Korea. We followed up the subjects until 7 years after the diagnosis of stroke without missing data because of the thorough nature of the NHIS. All kinds of cancers were included, which demonstrates organ specificity. In addition, the hypothetical influence of chemotherapy on ischemic stroke incidence was validated by subgroup analysis.

Our study showed that stroke risk increased in the cancer group compared with the control group, the risk was significantly high in the first 3 years, and its difference of stroke risk remained constant during the follow-up period such as a legacy effect. The risk of stroke was different according to organs where cancers originated from. Chemotherapy was independently associated with increased risk of ischemic stroke.

## Availability of Data and Materials

Because of the policy of National Health Insurance Service of Korea, dataset are not permitted to be taken out of the NHIS.

## Author Contributions

W-KS and JL supervised the project. H-SJ, JC, JS, J-WC, OB, G-MK, W-KS, and JL designed the study. JC, W-KS, and JL acquired and analyzed the data. H-SJ, JS, J-WC, OB, and G-MK interpreted the results. H-SJ and JC drafted the manuscript, and JS, J-WC, OB, G-MK, W-KS, and JL made critical revision of the manuscript.

### Conflict of Interest Statement

W-KS received honoraria for lectures from Pfizer, Sanofi-Aventis, Otsuka Korea, Dong-A Pharmaceutical Co., Ltd, Beyer, Daewoong pharmaceutical co. Ltd., Daiichi Sankyo Korea Co., Ltd. Boryung pharmaceutical, study grant from Daiichi Sankyo Korea Co., Ltd., and consulting fee from OBELAB Inc. The remaining authors declare that the research was conducted in the absence of any commercial or financial relationships that could be construed as a potential conflict of interest.
