# Supplementary material for: The Long-Term Effect of Cancer on Incident Stroke: A Nationwide Population-Based Cohort Study in Korea
Source: Front Neurol. 2019 Feb 5;10:52. doi: 10.3389/fneur.2019.00052 (PMC6370617; doi:10.3389/fneur.2019.00052)
Supplement: Supplementary file 1 [file Table_1.DOCX]

Supplemental data

**The long-term effect of cancer on incident stroke:**

**a nationwide population-based cohort study in Korea**

**Contents**

**Supplemental methods**

I. Operational definition of outcomes and vascular risk factors

**Supplemental Tables**

Supplemental Table 1. Cumulative incidence of each event according to time since index date

Supplemental Table 2. Risk of stroke by time since first cancer diagnosis

Supplemental Table 3. Adjusted relative hazards of each event by time interval in subjects with or without cancer

Supplemental Table 4. Stroke by cancer subtype among patients with cancer

Supplemental Table 5. Characteristics of subjects with ever chemotherapy and those without chemotherapy

**Supplemental methods**

1. Operational definition of outcomes and vascular risk factors

a) Stroke was diagnosed when the followed criteria were fulfilled:

(1) Newly registered diagnosis of ICD-10 code (I60–I63)

(2) Hospital admission of ≥3 days

(3) Acquisition of brain imaging including brain computed tomography or magnetic resonance imaging

b) Hypertension was identified when the subject had an ICD-10 code of I10–I15 or R030 or had a history of antihypertensive medication use.

c) Diabetes mellitus was identified when the subject had an ICD-10 code of E10–E14 or R739 or had a history of antidiabetic medication use.

d) Dyslipidemia was identified when the subject had an ICD-10 code of E78.0–E78.9 or had a history of antidyslipidemia medication use.

e) Heart failure was identified when a patient had an ICD-10 code of I50.

f) Atrial fibrillation was defined when the patient had an ICD-10 code of I48.

**Supplemental Tables**

Supplementary Table 1. Cumulative incidence of each event according to time since index date

| Time since index date, y | Any stroke | |  | Ischemic stroke | |  | Hemorrhagic stroke | |
| --- | --- | --- | --- | --- | --- | --- | --- | --- |
|  | Cancer | ^*^Matched |  | Cancer | Matched |  | Cancer | Matched |
| 1 | 0.86 (0.74, 0.99) | 0.31 (0.24, 0.39) |  | 0.80 (0.69, 0.93) | 0.27 (0.21, 0.35) |  | 0.08 (0.05, 0.13) | 0.05 (0.03, 0.09) |
| 2 | 1.37 (1.22, 1.53) | 0.72 (0.61, 0.84) |  | 1.23 (1.09, 1.39) | 0.63 (0.53, 0.75) |  | 0.17 (0.12, 0.23) | 0.11 (0.07, 0.16) |
| 3 | 1.90 (1.72, 2.09) | 1.17 (1.03, 1.33) |  | 1.71 (1.54, 1.89) | 1.05 (0.92, 1.19) |  | 0.24 (0.18, 0.31) | 0.17 (0.12, 0.23) |
| 4 | 2.28 (2.08, 2.49) | 1.67 (1.50, 1.85) |  | 2.05 (1.87, 2.25) | 1.48 (1.32, 1.65) |  | 0.30 (0.23, 0.38) | 0.26 (0.19, 0.33) |
| 5 | 2.71 (2.49, 2.93) | 2.10 (1.91, 2.30) |  | 2.44 (2.24, 2.66) | 1.85 (1.67, 2.04) |  | 0.36 (0.28, 0.45) | 0.34 (0.27, 0.43) |
| 6 | 3.05 (2.83, 3.29) | 2.54 (2.33, 2.76) |  | 2.76 (2.55, 2.99) | 2.23 (2.04, 2.44) |  | 0.41 (0.33, 0.50) | 0.41 (0.33, 0.50) |
| 7 | 3.43 (3.18, 3.68) | 3.04 (2.81, 3.28) |  | 3.10 (2.87, 3.35) | 2.67 (2.45, 2.89) |  | 0.46 (0.38, 0.57) | 0.50 (0.41, 0.60) |

^*^Matched to control

Supplemental table 2. Risk of ischemic stroke in subjects with and without cancer by baseline smoking statuses in subgroup with smoking data

| Stroke | Ex-smoker or current smoker at baseline  (n = 119,490) | | | |  | Non-smoker at baseline  (n = 247,856) | | | | p-value  for interaction |
| --- | --- | --- | --- | --- | --- | --- | --- | --- | --- | --- |
|  | Subjects with cancer  (n = 4,099) |  | Subjects without cancer  (n = 115,391) |  |  | Subjects with cancer  (n = 9,443) |  | Subjects without cancer  (n = 238,413) |  |  |
| Cumulative incidence (%) (95% CI) | 1.31 (0.97–1.73) |  | 0.36 (0.32–0.39) |  |  | 0.88 (0.70–1.11) |  | 0.38 (0.35–0.40) |  |  |
| Hazard ratio |  |  |  |  |  |  |  |  |  |  |
| Cause-specific HR (95% CI) |  |  |  |  |  |  |  |  |  |  |
| Unadjusted | 3.90 (2.88–5.29) |  | 1.0 |  |  | 2.43 (1.91–3.08) |  | 1.0 |  | 0.0161 |
| Adjusted^*^ | 1.12 (0.82–1.52) |  | 1.0 |  |  | 1.03 (0.81–1.32) |  | 1.0 |  | 0.6895 |
| Subdistribution HR (95% CI) |  |  |  |  |  |  |  |  |  |  |
| Unadjusted | 3.45 (2.54–4.68) |  | 1.0 |  |  | 2.22 (1.75–2.82) |  | 1.0 |  | 0.0261 |
| Adjusted^*^ | 0.92 (0.68–1.26) |  | 1.0 |  |  | 0.86 (0.68–1.10) |  | 1.0 |  | 0.7347 |

^*^Adjusted by sex, age group, hypertension, diabetes mellitus, dyslipidemia, coronary artery disease, heart failure, atrial fibrillation, antiplatelet agent, anticoagulant, and statin use

Supplemental Table 3. Adjusted relative hazards of each event by time interval in subjects with or without cancer

| y | Original cohort | | | | | | | |
| --- | --- | --- | --- | --- | --- | --- | --- | --- |
|  | Any stroke | |  | Ischemic stroke | |  | Hemorrhagic stroke | |
|  | SubHR^*^ (95% CI) | P |  | SubHR (95% CI) | P |  | SubHR (95% CI) | P |
| Original cohort | | |  |  |  |  |  |  |
| 0–1 | 2.45 (2.06, 2.92) | <0.0001 |  | 2.61 (2.18, 3.12) | <0.0001 |  | 1.58 (0.92, 2.72) | 0.0996 |
| 1–2 | 1.39 (1.13, 1.70) | 0.0018 |  | 1.28 (1.02, 1.60) | 0.0312 |  | 1.72 (1.05, 2.81) | 0.0317 |
| 2–3 | 1.43 (1.17, 1.76) | 0.0005 |  | 1.50 (1.21, 1.86) | 0.0002 |  | 0.98 (0.57, 1.71) | 0.9538 |
| 3–4 | 1.04 (0.82, 1.31) | 0.7423 |  | 1.05 (0.82, 1.35) | 0.6797 |  | 0.94 (0.53, 1.68) | 0.8407 |
| 4–5 | 1.15 (0.92, 1.44) | 0.2093 |  | 1.19 (0.94, 1.50) | 0.1501 |  | 1.09 (0.61, 1.94) | 0.7792 |
| 5–6 | 0.92 (0.72, 1.17) | 0.4868 |  | 0.98 (0.76, 1.26) | 0.8484 |  | 0.75 (0.39, 1.44) | 0.3917 |
| 6–7 | 0.97 (0.77, 1.23) | 0.8067 |  | 0.99 (0.78, 1.27) | 0.9638 |  | 0.96 (0.53, 1.73) | 0.8839 |
| Matched cohort | | |  |  |  |  |  |  |
| 0–1 | 2.78 (2.08, 3.70) | <0.0001 |  | 2.97 (2.20, 4.03) | <0.0001 |  | 1.70 (0.78, 3.72) | 0.1828 |
| 1–2 | 1.37 (1.03, 1.82) | 0.0300 |  | 1.30 (0.96, 1.77) | 0.0891 |  | 1.64 (0.79, 3.41) | 0.1840 |
| 2–3 | 1.33 (1.01, 1.76) | 0.0394 |  | 1.31 (0.98, 1.75) | 0.0642 |  | 1.22 (0.58, 2.60) | 0.6015 |
| 3–4 | 0.90 (0.67, 1.21) | 0.4904 |  | 0.93 (0.68, 1.27) | 0.6431 |  | 0.77 (0.37, 1.60) | 0.4886 |
| 4–5 | 1.16 (0.86, 1.55) | 0.3376 |  | 1.23 (0.90, 1.68) | 0.1998 |  | 0.85 (0.42, 1.73) | 0.6537 |
| 5–6 | 0.95 (0.70, 1.30) | 0.7605 |  | 1.01 (0.73, 1.40) | 0.9514 |  | 0.91 (0.40, 2.08) | 0.8279 |
| 6–7 | 0.89 (0.66, 1.19) | 0.4215 |  | 0.93 (0.69, 1.27) | 0.6592 |  | 0.76 (0.37, 1.56) | 0.4486 |

^*^Subdistribution hazard ratio: All-cause mortality was considered a competing risk event.

Adjusted variables include sex, age, hypertension, diabetes mellitus, dyslipidemia, coronary artery disease, heart failure, artrial fibrillation, and medication use (anticoagulant, antiplatelet agent, statin).

Supplementary Table 4. Stroke by cancer subtype among patients with cancer

| ICD-10 code | Code of first diagnosed cancer (by organ) | Total (N = 20,707) | Number of events within 7 years | | | |
| --- | --- | --- | --- | --- | --- | --- |
|  |  | n (%) | Any stroke | Ischemic stroke | Hemorrhagic stroke | Death |
| C00–C14 | Lip, oral cavity, and pharynx | 1,065 (5.1) | 50 | 48 | 4 | 157 |
| C15–C26 | Digestive organs | 9,033 (43.6) | 306 | 276 | 42 | 2,255 |
| C30–C-39 | Respiratory and intrathoracic organs | 2,096 (10.1) | 89 | 80 | 13 | 787 |
| C40–C41 | Bone and articular cartilage | 99 (0.5) | 5 | 4 | 1 | 18 |
| C43–C44 | Melanomas and soft tissue | 229 (1.1) | 8 | 6 | 2 | 45 |
| C69–C72 | Eye, brain, and central nervous system | 297 (1.4) | 16 | 14 | 2 | 64 |
| C73–C75 | Thyroid and other endocrine glands | 1,433 (6.9) | 14 | 12 | 3 | 38 |
| C76–C80 | Ill-defined, secondary, and unspecified sites | 380 (1.8) | 14 | 11 | 3 | 135 |
| C81–C96 | Lymphoid, hematopoietic, and related tissue | 134 (0.6) | 2 | 2 | 0 | 34 |
| C64–C68 | Urinary tract | 665 (3.2) | 20 | 17 | 4 | 142 |
| C76–C80 | Ill-defined, secondary, and unspecified sites | 885 (4.3) | 31 | 30 | 3 | 343 |
| C50–C58 | Breast, female or male reproductive organs, etc. | 4,391 (21.2) | 154 | 142 | 19 | 527 |

Supplemental table 5. Characteristics of subjects with ever chemotherapy and those without chemotherapy

|  | Ever chemotherapy^*^ | | Never chemotherapy | |  |
| --- | --- | --- | --- | --- | --- |
|  | (n=6,211) | | (n=14,496) | | p-value(a) |
| Age group |  |  |  |  | <.0001 |
| 19–30 years | 117 (1.9) | | 730 (5.0) | |  |
| 30–40 years | 457 (7.4) | | 1,665 (11.5) | |  |
| 40–50 years | 1,059 (17.1) | | 3,020 (20.8) | |  |
| 50–60 years | 1,312 (21.1) | | 3,183 (22.0) | |  |
| 60–70 years | 1,645 (26.5) | | 2,968 (20.5) | |  |
| 70–80 years | 1,290 (20.8) | | 2,083 (14.4) | |  |
| 80 years | 331 (5.3) | | 847 (5.8) | |  |
|  |  |  |  |  |  |
| Sex |  |  |  |  | 0.4471 |
| Men | 3,197 (51.5) | | 7,378 (50.9) | |  |
| Women | 3,014 (48.5) | | 7,118 (49.1) | |  |
|  |  |  |  |  |  |
| Comorbidities |  |  |  |  |  |
| Hypertension | 3,942 (63.5) | | 8,743 (60.3) | | <.0001 |
| Diabetes | 4,057 (65.3) | | 9,014 (62.2) | | <.0001 |
| Dyslipidemia | 4,224 (68.0) | | 10,514 (72.5) | | <.0001 |
| Coronary artery disease | 2,517 (40.5) | | 5,680 (39.2) | | 0.0704 |
| Heart failure | 830 (13.4) | | 1,873 (12.9) | | 0.3863 |
| Atrial fibrillation | 512 (8.2) | | 976 (6.7) | | 0.0001 |
|  |  |  |  |  |  |
| Concurrent medication |  |  |  |  |  |
| Anticoagulant | 302 (4.9) | | 457 (3.2) | | <.0001 |
| Antiplatelet agents | 967 (15.6) | | 2,460 (17.0) | | 0.0129 |
| Statin | 2,099 (33.8) | | 5,656 (39.0) | | <.0001 |

*Those who were prescribed for chemotherapeutic agents at least once p-value by chi-square test
